# Effects of transcutaneous auricular vagus nerve stimulation and exploration of brain network mechanisms in children with high−functioning autism spectrum disorder: study protocol for a randomized controlled trial

**DOI:** 10.3389/fpsyt.2024.1337101

**Published:** 2024-02-05

**Authors:** Ke Sun, Ying Li, Zhenhang Zhai, Heqing Yin, Shuli Liang, Feng Zhai, Yonghua Cui, Guojun Zhang

**Affiliations:** ^1^ Functional Neurosurgery Department, National Center for Children’s Health, Beijing Children’s Hospital, Capital Medical University, Beijing, China; ^2^ Department of Psychiatry, Beijing Children’s Hospital, Beijing, China

**Keywords:** high-functioning autism, transcutaneous auricular vagus nerve stimulation, neuromodulation, protocol, randomized control trial

## Abstract

**Background:**

Autism Spectrum Disorders (ASD) are a collection of neurodevelopmental diseases characterized by poor social interaction and communication, a limited range of interests, and stereotyped behavior. High-functioning autism (HFA) indicates a subgroup of individuals with autism who possess cognitive and/or language skills that are within the average to above-normal range for their age. Transcutaneous auricular vagus nerve stimulation (taVNS) holds promise in children with HFA. However, few studies have used randomized controlled trials to validate the effectiveness of taVNS. Therefore, in this study, we intend to provide a study protocol to examine the therapeutic effects of taVNS in individuals diagnosed with HFA and to investigate the process of brain network remodeling in individuals with ASD using functional imaging techniques to observe alterations in large-scale neural networks.

**Methods and design:**

We planned to employ a randomized, double-blind experimental design, including 40 children receiving sham stimulation and 40 children receiving real stimulation. We will assess clinical scales and perform functional imaging examinations before and after the stimulation. Additionally, we will include age- and gender-matched healthy children as controls and conduct functional imaging examinations. We plan first to observe the therapeutic effects of taVNS. Furthermore, we will observe the impact of taVNS stimulation on the brain network.

**Discussion:**

taVNS was a low-risk, easy-to-administer, low-cost, and portable option to modulate the vagus system. taVNS may improve the social performance of HFA. Changes in the network properties of the large-scale brain network may be related to the efficacy of taVNS.

**Clinical trial registration:**

http://www.chictr.org.cn, identifier ChiCTR2300074035.

## Introduction

Autism Spectrum Disorders (ASD) are a collection of neurodevelopmental diseases characterized by poor social interaction and communication, a limited range of interests, and stereotyped behavior. (American Psychiatric Association, 2013) (1). Its prevalence has been rising gradually. According to the most recent survey conducted by the Centers for Disease Control and Prevention (CDC) ‘s Autism and Developmental Disabilities Monitoring (ADDM) Network in 2020, it is estimated that approximately 1 in 36 children had been diagnosed with ASD. The disorder affects all races and countries and has drawn tremendous attention (2).

ASD is a heterogeneous disorder. Even among individuals who meet the core diagnostic criteria for ASD, there is significant variability in intelligence (3). Low intelligence is often concomitant with ASD, and these patients are considered as low functioning autism. On the other side, patients with average or even above-normal cognitive and/or language skills are classified as high-functioning autism (HFA) (4). Patients with HFA usually have an IQ of at least 70 and demonstrate near-normal levels of language development (4, 5). However, they still exhibit notable deficits in socio-emotional function, language comprehension, and expressive abilities (6, 7). These sustained deficiencies in social behavior have wide-ranging consequences on the general functioning of the patients and hinder participation in meaningful occupations and important roles in society (4, 8, 9). It is worth noting that only a small percentage, ranging from 5% to 15%, of adults with ASD can achieve a relatively normal social life and acceptable levels of academic or occupational functioning (10). Additionally, few individuals with ASD attain enough independence to pursue marriage or homeownership (4).

Currently, there are no approved medications able to address the core symptoms of ASD, which include social communication difficulties as well as limited restricted and repetitive behaviors and interests (11). Available pharmacological treatments aim only to alleviate concomitant symptoms, including inattention, compulsions, hyperactivity, anxiousness, sleep disruption, and irritation (12). Applied Behavior Analysis (ABA), an evidence-based approach, is shown to be potentially beneficial in enhancing certain outcomes for individuals with ASD. However, it can be administered only by professional agencies, making it time-consuming, costly, and difficultly available (13, 14). Therefore, exploring innovative, effective, and applicable treatments for children with ASD is of great importance.

Neuromodulation has been applied successfully in treating many psychiatric diseases because of its unique advantage (15). Vagus nerve stimulation (VNS) is an effective neuromodulation therapy that received initial approval from the US Food and Drug Administration (FDA) for drug-resistant epilepsy in 1997 (16). After the discovery of the mood-altering effects of VNS, its use expanded to treat treatment-resistant depression and was approved by the FDA in 2005 (17, 18). In patients with intractable epilepsy and ASD, VNS not only provides seizure control but also alleviates symptoms of ASD, which implies a potential therapeutic effect of VNS for ASD (19).

However, the invasive nature of VNS has limited its usage in certain diseases. Transcutaneous auricular vagus nerve stimulation (taVNS) stimulates the ear directly with a non-invasive method (15, 20, 21), and it had a higher potential application value in ASD because of an omission of surgical intervention procedures. Studies have shown that taVNS can yield a comparative effect to traditional VNS, and the effect is primarily based on the anatomical fact that the ear is the sole site on the human body where the afferent vagus nerve distributes (22).

Social dysfunction is a prominent characteristic of ASD, encompassing impairments in emotion recognition, interception accuracy, and emotional memory (23). Prior studies have demonstrated that taVNS can enhance various aspects of social cognition, including social attention (24), social-emotional understanding and regulation (25), interception accuracy (26), and social cognitive flexibility (27). Therefore, taVNS may be a promising alternative treatment for individuals with ASD, offering potential improvements in social cognition. Notably, in an open-label trial of 12 individuals with ASD treated with taVNS, average scores for anxiety and sleepiness were improved (28). Inspired by the results of this preliminary trial, a more rigorous study is warranted to provide further validation of the therapeutic efficacy of taVNS in individuals with HFA.

ASD is widely acknowledged as a neurodevelopmental disorder affecting brain networks (29, 30). Previous neuroimaging studies have indicated that individuals with ASD exhibit atypical brain structure and connectivity, particularly in regions associated with social cognition, emotional processing, and language abilities (30, 31). Similar results have been found in patients with HFA (32, 33). Previous studies have demonstrated that taVNS exerts modulatory effect on cortical and subcortical areas of the brain in healthy adults (34) and aiding in the regulation of socio-emotional functions impaired in depression (35). Furthermore, the neuroplasticity of children’s brains is significant (36), and taVNS has the potential to enhance it further by modulating brain networks. However, there is currently a lack of studies investigating the impact of taVNS on brain network modification in children with HFA.

Based on the aforementioned evidence, we designed our protocol with two primary objectives: First, it aims to examine the therapeutic effects of taVNS in individuals diagnosed with HFA; Second, it intends to investigate the process of brain network remodeling after taVNS using functional imaging techniques. We will use graph-theoretic analysis to compare the brain network characteristics of individuals with HFA before and after receiving taVNS. Additionally, we will examine the differences in these characteristics between patients with HFA and healthy controls, considering both structural and functional connectivity perspectives. Our hypothesis posits that taVNS has the potential to enhance the efficiency and flexibility of brain networks in HFA patients, leading to an improvement in their social cognitive abilities.

## Methods/design

### Study design

**Figure 1:**
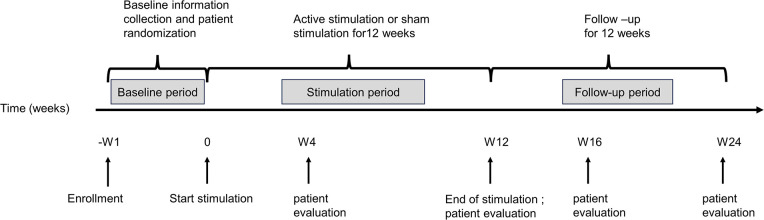
A schematic representation of the study timeline of events.

The study will be a randomized, double-blind, sham-controlled clinical trial, which will include patients assigned to active taVNS and sham taVNS groups and healthy subjects assigned to a healthy control group (HC). The study timeline of events is listed in **Figure 1**. For included patients, this study consisted of three basic phases: (a) a one-week baseline; (b) a consecutive 12-week treatment period without weekend interruption (Week 1 - Week 12, W1-W12); and (c) a follow-up period of 12 weeks (Week 13 – Week 24, W13 - W24). After enrollment, an interview will be used to acquire general patient information, including age, gender, duration of HFA, family history, and results of the clinical assessment tools during the baseline. Following the baseline period, patients will be assigned to one of the two treatment groups randomly to receive active taVNS or sham taVNS treatment using a random number table, which will be generated by SPSS 25.0 before enrollment of the first patient. During the follow-up period, patients will be evaluated with clinical assessment tools as well as structural and functional imaging scans.

The treatment condition assignment will remain undisclosed to patients and independent observers who perform clinical assessments and MRI scan technicians until the last interview. Only the technicians performing the taVNS procedure and the physician assigning the randomization without patient contact will be aware of the group information. No communication will be allowed between patients during any phase of the study to maintain blindness.

**Figure 2:**
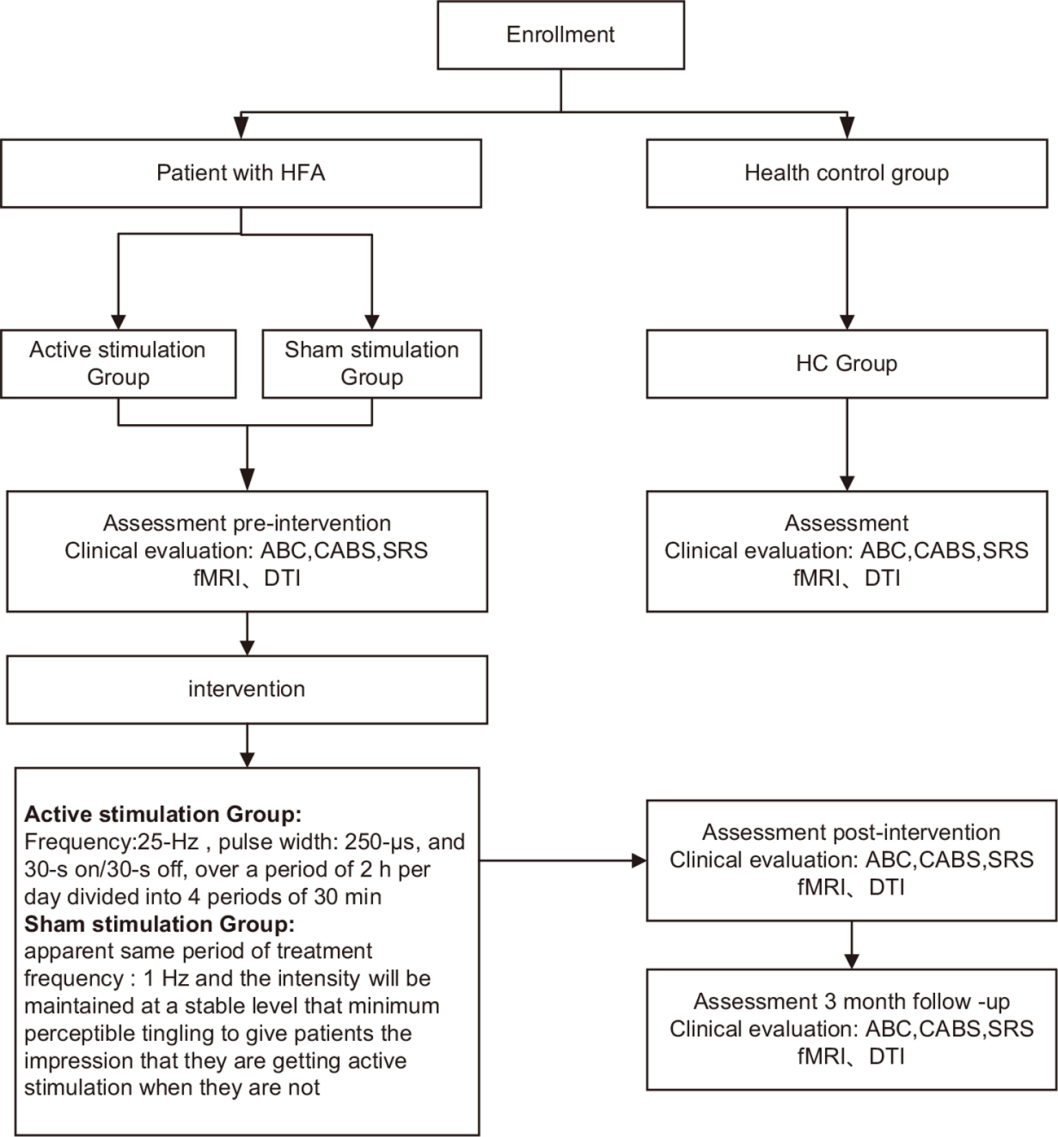
Proposed study procedures and participant flow. HFA, high−functioning autism spectrum disorder; ABC, Autism Behavior Scale; CABS, Clancy Autism Behavioral Scale; SRS, Social Responsiveness Scale; HC, healthy controls; fMRI 、DTI.

Patients will be evaluated using the clinical assessment tools at five time points (baseline; W4, the first month of treatment; W12, right after treatment; W16, short-term follow-up; W24, long-term follow-up) (**Table 1**). MRI scan will be acquired at three time points (baseline, W12, and W24). More details regarding the clinical procedures can be found in **Figure 2**; **Table 1**. Healthy subjects in the HC group will be evaluated with the same clinical tools and undergo an MRI scan only once. The clinical endpoints are assessed by blinded observers who are not involved. Furthermore, all dropouts will be documented and reported, specifying the reasons for their withdrawal.

**Table 1:** Measurements at each time point.

Measurements/time point	Recruitment	Baseline	4 weeks	12 weeks	16 weeks	24 weeks
Inclusion/exclusion criteria	√	N/A	N/A	N/A	N/A	N/A
Behavioral Checklist for Children with High-Functioning Autism/Asperger’s Disorder	N/A	√	√	√	√	√
ABC	N/A	√	√	√	√	√
CABS	N/A	√	√	√	√	√
SRS	N/A	√	√	√	√	√
WISC	N/A	√	√	√	√	√
MRI scanning	N/A	√	N/A	√	√	√

√, need to be finished; ABC, Autism Behavior Checklist; CABS, Clancy Autism Behavioral Scale; SRS, Social Responsiveness Scale; WISC, Wechsler Intelligence Scale. N/A, Not applicable.

This study is authorized by the ethical committee of Beijing Children’s Hospital and will be conducted in compliance with the Declaration of Helsinki. Informed written consent will be acquired from the children and/or their legal guardians.

### Participants

#### Recruitment of participants

The Beijing Children’s Hospital Department of Psychiatry will recruit participants with HFA diagnoses from clinical practice, and a clinical multidisciplinary team involving two experienced psychiatrists will diagnose patients according to the DSM-5 diagnostic criteria. The inclusion criteria are as follows:

- the age range is between 6-12 years old.
- Wechsler intelligence test IQ of at least 80 for Chinese children.
- ability and willingness to cooperate with relevant assessments and tests.
- right-handedness.

The exclusion criteria include:

- seizure disorder or other neurological diseases (including brain tumors and craniocerebral trauma).
- concomitant with other mental disorders, except for attention deficit hyperactivity disorder (ADHD) and obsessive-compulsive disorder (OCD).
- claustrophobia.
- impulsivity or aggression.
- presence of metal products in the body.

Discontinuation and withdrawal criteria:

- sudden acute illness who are unable to progress to the next phase in the study.
- poor adherence, and biased results will be regarded as withdrawing from the study.
- other uncontrollable factors or self-withdrawal from the study.

In this study, recruitment is set to occur over a period of one and a half years (from August 2023 to December 2024). Beijing primary schools will be recruited to provide healthy controls meeting the following inclusion criteria:

- The age range is between 6-12 years old.
- IQ of at least 80.
- right-handedness.

Exclusion criteria include:

- diagnosis of neurological or mental disorders.
- claustrophobia.
- presence of metal products in the body.

#### Sample size estimation

Power Analysis and Sample Size Software version 15.0 (PASS, NCSS Statistical Software, Kaysville, UT, USA) was used to determine the study’s sample size. An alpha of 0.05 and a power of 0.9 are used in the study. Based on preliminary trial results, the Clancy Autism Behavioral Scale (CABS) score indicates a curative effect, so the mean and standard deviation of the paired differences are 5 and 8, respectively (15). The PASS software calculation resulted in a minimum requirement of 34 participants per group, accounting for an estimated 20% dropout rate. Considering a 15% rate of invalid MRI data based on previous experience, a minimum of 40 subjects will be recruited per group. Therefore, 40 patients will be enrolled in each group, totaling 80 patients for the study.

### Assessment instruments

#### Behavioral checklist for children with high-functioning autism/Asperger’s disorder high-functioning autism checklist

The Behavioral Checklist for Children with High-Functioning Autism, developed by Jung-Fen Chang, will be utilized in this study. This scale consists of two parts: the first is a primary survey, while the second is a 60-question quiz for reference purposes. The questions are categorized into three groups- social, communication, and behavioral - to evaluate Asperger’s disorder in these domains (37).

#### Autism behavior checklist

The ABC was initially established by Krug et al. in the United States in 1978 and subsequently validated in 1980 to determine its psychometric properties, demonstrating a reliability coefficient of 0.87 (38). The checklist comprises 57 elements categorized into five groups: sensory, relational, body and object usage, language, and socialization/self-help. Scores are based on a four-point frequency scale, with higher scores indicating more abnormal behavior. Each sub-scale’s total score can be calculated by calculating the domain fractions and overall fractions. These scores provide an indication of the severity of the behavioral characteristics associated with autism, with higher scores reflecting more prominent features (38). It is commonly implemented for screening individuals aged 3-35. ABC Scale has gained popularity as a screening tool for autism spectrum disorder due to its user-friendly nature, ease of scoring, and cost-effectiveness.

#### CABS

The CABS is a parent-reported autism diagnostic scale compiled by Clancy in 1969 comprising 14 entries. Tse Ching Fen et al. revised the scale in Taiwan in 1983, replacing the original 2-point method with three types of reaction strength: “never 0,” “occasionally 1-point,” and “often 2-point”. A total score of ≥14 serves as the preliminary screening criteria for autism, while a total score of ≥14 points combined with a “never” item score of <3. Moreover, a “regular” item score of ≥6 can be used to diagnose autism (39). The CABS scale is presently regarded as the most extensively utilized autism screening tool in Mainland China.

#### Social responsiveness scale

Children with autism aged 4-18 are routinely tested by the SRS. Social contact, social cognition, social interest, social motivation, and daily routines are among the 65 items in the SRS that describe children’s daily social experiences. Studies have examined the use of the SRS for screening autism spectrum disorders in children over four years of age. In 2017, Cen et al. evaluated the use of the SRS scale in China (40). The study found that the scale demonstrated excellent reliability and validity, supporting its usage in clinical and related research. Li et al. assessed the reliability and validity of the SRS scale in 275 children in 2018. The results indicated 59.8% sensitivity, 77.5% specificity, and 88.4% positive predictive value (41).

#### Wechsler intelligence scale

The Wechsler Intelligence Scale for Children’s Chinese version will be used to assess the participants’ intelligence (26). The test follows a national norm in urban and rural editions. Test-retest reliability of 0.59 to 0.86 is shown by a split-half correlation coefficient of 0.8 and an IQ split-half reliability of 0.9 (42). Additionally, the scale has been validated structurally. This tool is widely recognized as an essential measure of children’s IQ levels in China.

### Interventions

Inclusion of patients with HFA will be randomized into one of two groups. For the active group, patients will be subjected to a 25 Hz stimulation frequency, 250 s pulse width, and a 30-s on/30-s off cycle for a total of 2 hours divided into four 30-minute periods. Patients remain quiet during each 30-minute session. The stimulation intensity will be adjusted to a level where patients experience the maximum tolerable intensity of pain (43–45). In contrast, the sham group will undergo a placebo procedure designed to mimic the active treatment condition. The sham group will be positioned anatomically identical to the active treatment group, receiving the same duration of treatment. However, the stimulation frequency will be set at 1 Hz, and the intensity level will only elicit the minimum perceptible tingling sensation, creating the illusion of active stimulation. The National Institutes for Food and Drug Control (NIFDC)-certified taVNS device TVNS-100 (Xinzhile, Jiangxi, China) will be utilized for the taVNS procedure, with electrodes placed on both the inner and outer surfaces of the tragus in bilateral ears (43). Patients and caregivers will receive professional instructions on how to operate the stimulator and then perform the stimulation by themselves routinely. In addition, patients and caregivers will be instructed to promptly monitor and report any potential side effects, such as headache, tinnitus, vertigo, or nausea, to their doctors during or after stimulation. Depending on the situation, the stimulation intensity may be adjusted, or the stimulation may be stopped.

### MRI scanning

#### MRI sequences

The MR750 3.0T MRI scanner (GE Healthcare, USA) will be utilized to scan all participants at Beijing Children’s Hospital’s Department of Radiology. A three-dimensional (3D) magnetization-prepared rapid gradient echo sequence with the following parameters will be used to obtain T1-weighted images: repetition time (TR)/echo time (TE)/inversion time (TI)=2530/2.34/1100 ms, flip angle = 7°, slice count = 176, section thickness = 1mm, matrix size = 256×256. The duration of the T1 sequence was 649 seconds. Subsequently, T2-weighted image scans lasting for 60 seconds will be performed on all participants to eliminate the possibility of incidental disorders of the central nervous system (46).

Functional image data (Resting-state fMRI, rs-fMRI)will be obtained using T2-weighted echo planar imaging with the following parameters: TR/TE =2500/21 ms, flip angle=90°, number of slices=42, slice thickness=3.5 mm, field of view (FOV)=20×20 cm^2^, matrix size =64×64, voxel size=3.1×3.1×3.5 mm^3^, and bandwidth=2.52 kHz/Px. An echo planar imaging sequence will be used to acquire the diffusion data, with parameters TR/TE =7000/70 ms, flip angle=90°, data matrix size =112×112, FOV= 20×20 cm^2^, slice thickness=2 mm, number of slices=60, and scanning time= 9 minutes and 40 seconds. For the 3D T1-weighted turbo field echo sequence and parameters will have the following parameters: TR/TE=12/5.9 ms, flip angle=8°, matrix size=256×256, FOV =25.6×25.6 cm^2^, slice thickness=1.6 mm with 0.8 mm gaps between slices, number of slices=180, and scanning time= 5 minutes and 56 seconds. All participants will be given clear instructions to keep their eyes closed during the scanning procedure while ensuring they remain awake.

#### MR preprocessing

Each participant’s rs-fMRI data will be preprocessed using Statistical Parametric Mapping software (SPM12) and a powerful toolbox called Data Processing & Analysis of Brain Imaging (DPABIV7.0). The following specific steps will be performed for each subject: converting the DICOM to NIFTI format, removing the first ten time points to exclude the effects of subject maladaptation and magnetic field inhomogeneity at the beginning of the scan, and leaving the remaining time points for further spatial and temporal alignment and head rotation correction. If the subject’s head movement is >2 mm in the translation range or >2 ° in rotation, the subject’s data should be excluded. Additionally, time and spatial normalization procedures will be conducted. The images will be aligned to the Montreal Neurological Institute (MNI) space, with each voxel measuring 3 × 3 × 3 mm³. Nuisance covariates, including the Friston 24 parameter model, as well as signals from cerebrospinal fluid and white matter, will be removed through regression analysis to correct the realigned data. A 4 mm full width at half-maximum isotropic Gaussian kernel was utilized for spatial smoothing, while linear signals were eliminated. Furthermore, a temporal band-pass filter will be applied, restricting the frequency range to 0.01–0.08 Hz (47). The diffusion tensor imaging data preprocessing will be conducted using the open-source software Pipeline for analyzing brain diffusion images (PANDA), which also allows for the construction of brain networks and can be downloaded at http://www.nitrc.org/projects/panda/. The preprocessing typically involves several key steps. First, the 3D T1 structural image will be co-registered with the b0 image to eliminate the effects of eddy current distortions and motion artifact; next, extraction of brain tissue from corrected data, removal of scalp and other non-brain tissue structures; the diffusion space will be subsequently aligned with the T1 template, utilizing the MNI space as a reference. Thus, the diffusion space will be mapped to the MNI space, facilitating the reconstruction of fibers connecting each pair of brain regions within this space. The whole-brain fibers will be constructed using a fiber assignment algorithm based on continuous tracking. The interregional connection density will be quantified as the fractional anisotropy (FA) value. Based on our experience, fiber tracking will cease at voxels where FA < 0.2 or when the angle between two consecutive voxel’s eigenvectors exceeds 35° (48).

### Network construction and analysis

#### Functional network

A brain network is composed of nodes and edges. We will also use the AAL template to construct the functional network to assign each brain region without the cerebellum as an anatomical region of interest (ROI). The ROIs will then be designated as nodes within the functional network, allowing us to extract the average time series for each node. An edge in a functional network is thought to be a functional connection between two brain areas. By calculating the Pearson correlation coefficient between the time series of each pair of ROI regions, we will obtain the functional connectivity matrix of each subject’s brain. It allows for a more complete understanding of the network organization since it contains additional information about the strength of functional connectivity on a continuous scale (49).

#### Structural network

The construction of structural networks based on diffusion imaging will also use AAL templates. Each brain region is referred to as a node, while detectable connections between nodes are considered edges. Two nodes are regarded as linked if at least one fiber bundle links them. For each subject, we calculate the weights of the edges using the connection density per unit of surface as its weights to obtain an undirected weighted structural connectivity network. Following that, the topological characteristics of the brain network will be assessed using graph theory. The thresholds for network groups will be quantified using a wide range of sparsity (0.05–0.5) with a 0.05 interval. To facilitate the study of the coupling between functional and structural connectivity networks, ensure structural connectivity networks are connected and have a minimum number of pseudo-edges (48).

#### Network analysis

The small-world properties, global properties, and local properties of brain networks will be analyzed using graph theory. There are five small-world properties: clustering coefficient (Cp), characteristic path length (Lp), normalized clustering coefficient (Gamma, γ), normalized characteristic path length (Lambda, λ), and small-worldness (Sigma, σ) (50, 51). A network is regarded to have small-world features when γ >> 1, λ ≈1, and σ > 1 (48, 52). Global network properties consist of global efficiency (Eg) and local efficiency (Eloc). Nodal properties, or local properties, include nodal efficiency (NE), betweenness centrality (BC), and nodal degree centrality (DC) (51, 53). A node’s NE can be measured as its capacity to transmit information to other nodes, its BC as its capacity to influence the flow of information between all other nodes, and its DC can be measured as the number of edges it connects with (52, 54).. In a previous publication, these network measures were defined in detail (54).

### Statistical analysis

Basic analyses will be conducted using SPSS 25.0 software. At baseline, independent samples t-tests will be used to determine if there is no statistical difference between the two intervention groups; paired samples t-tests will then be used to examine the differences between mean scores before and after the taVNS intervention to determine the effectiveness of the treatment. Additionally, for the imaging study, we will use two-sample t-tests to compare brain network parameters between the HC and sham groups, as well as paired-sample t-tests to determine brain network parameters between the sham and active groups. Based on age and gender as covariates, a two-sample t-test will be used to compare the small-world properties and global parameters of the anatomical network between the HC group and the sham group. The level of significance for all statistical tests is set at p< 0.05. A Bonferroni correction will be performed on the t-tests utilized to examine group differences in behavioral and brain measurements, to account for multiple comparisons.

### Clinical trial registry

Beijing Children’s Hospital, Capital Medical University, Ethics Committee approved this randomized controlled trial. The participant and his or her guardian will sign and submit an informed consent form. If participants feel unwell, they can withdraw from the study at any time, and they will be referred to a follow-up doctor for further examination and treatment. The trial has been registered on the Chinese Clinical Trial Registry with the registration number ChiCTR2300074035 (http://www.chictr.org.cn).

## Discussion

This protocol presents a study that evaluates the efficacy of taVNS in children with HFA and explores the associated cerebral network change. To our knowledge, this study may provide new evidence about novel non-pharmacological interventions for ASD. The protocol also aims to examine multiple aspects of the treatment response, going beyond the impact of taVNS on psychosocial functioning and neurophysiological mechanisms.

The treatment approach for ASD typically involves behavioral therapies aimed at reducing problem behaviors and general distress while enhancing social and communication skills. However, these treatments are often unsatisfactory due to high rates of nonresponse, relapse, and occurrence of intolerable side effects (12). Therefore, the exploration of using taVNS to treat ASD, as will be done in our study, will shed light on finding alternative strategies for ASD.

It is currently recognized that there is dysregulation of the sympathetic nervous system in children with ASD, and the vagal tone is low. Decreased vagal activity is associated with the behavioral and linguistic changes present in autism, while higher vagal activity has been linked to improved communication abilities (55). The vagus nerve coordinates the function of the bronchi, lungs, heart, and esophagus (22), regulates the autonomic nervous system, participates in the socio-emotional processes, and also has a role in adaptive behavior (56). Hence, stimulation of the vagus nerve, as in VNS, can change the vagal tone and re-regulate organism function, increase pathologically inadequate vagal response, and modulate the parasympathetic nervous system’s activities in patients with neurodevelopmental disorders (44, 55).

Recently, VNS has been utilized in children with ASD who have co-occurring epilepsy or epileptiform discharges on EEG, demonstrating therapeutic benefits for alleviating clinical symptoms (57). A study involving six children diagnosed with tuberous sclerosis complex and intellectual disability found that one child with ASD exhibited an over 90% decrease in seizure frequency and an 80% decrease in injurious behaviors concurrently when undergoing VNS (46). Hu et al. demonstrated that VNS stimulation led to increased vagal activity, resulting in improved social skills and parent-child interactions and positive socioemotional outcomes for children with ASD (46). Wang et al. found that VNS improved linguistic and cognitive performance in children with ASD (19). Thus, VNS may serve as a potential adjunctive intervention for children with ASD.

There is only one branch of the vagus nerve on the surface of the body, the auricular branch (20). In neuropsychiatric disorders, taVNS stimulation of the auricular branch has gained significant attention. While the mechanisms of taVNS remain unclear, studies have discovered its effects in modulating socially relevant emotional and visceral states. ASD is a neurodevelopmental disorder involving structural abnormalities in several brain regions, including the supplementary motor area, somatosensory cortex, dorsal prefrontal cortex, premotor cortex, hippocampus, and basal ganglia (58). More importantly, evidence suggests that taVNS can activate brain regions associated with ASD, with electrical stimulation possibly propagating through a reverse pathway from peripheral nerves to the brainstem and central structures. This process may induce therapeutic effects by leveraging the “bottom-up” mechanisms of the central nervous system (21).

Functional nuclear magnetic resonance imaging studies of taVNS indicate enhanced blood oxygenation level-dependent signals in the amygdala, thalamus, and frontal cortex (59). It is consistent with brain activity modulation by implantable VNS. Furthermore, stimulation of the auricular tegmentum activates the amygdala and solitary fasciculus to a greater degree (59). These nuclei and brain regions may have close associations with brain regions experiencing functional deficits in individuals with HFA. In this study, patients will undergo clinical and imaging assessments during the baseline period. Additionally, clinical evaluations will be conducted during the short-term treatment period (W4), and clinical observations and neuroimaging investigations will be carried out immediately after treatment (W12), during the early follow-up period (W16), and throughout the late follow-up period (W24). This comprehensive approach enables a thorough examination of the therapeutic effects of taVNS on patients with HFA during the early stages of treatment, as well as the establishment of relatively stable brain network changes induced by taVNS. Furthermore, follow-up observations may shed light on the long-term stability of the taVNS-induced brain network alterations. Hopefully, with the most recent advances mentioned above, it is anticipated to uncover the effectiveness of taVNS and the underlying mechanisms with structural and functional magnetic resonance imaging in our study.

## Limitations

There are three limitations in this study. First, the sample size of this study may be sufficient to provide preliminary insights; however, it may not represent the entire population so generalizability may be limited. Second, the research was conducted at a single center. However, further observations utilizing taVNS in larger populations of children with HFA across multiple centers are certainly warranted. In addition, psychiatrists in our hospital have not received training in the use of Autism Diagnostic Observation Schedule-Second Edition (ADOS-2) and Autism Diagnostic Interview-Revised (ADI-R). Consequently, they are not certified to conduct evaluations using ADOS-2 and ADI-R.

## Conclusion

In light of the results of this study, it will be possible to optimize non-invasive neuromodulation therapies for children with HFA. Additionally, the results will shed light on how taVNS improves social skills in children with HFA. Our understanding of taVNS mechanisms and the pathogenesis of HFA will also be enhanced through the study of brain networks.

## Ethics statement

The studies involving humans were approved by ethics committee of Beijing Children’s Hospital. The studies were conducted in accordance with the local legislation and institutional requirements. Written informed consent for participation in this study was provided by the participants’ legal guardians/next of kin.

## Author contributions

KS: Methodology, Writing – original draft. YL: Methodology, Writing – review & editing. ZZ: Investigation, Writing – review & editing. HY: Methodology, Writing – review & editing. SL: Investigation, Methodology, Writing – review & editing. FZ: Writing – review & editing, Investigation, Methodology. YC: Conceptualization, Writing – review & editing. GZ: Conceptualization, Writing – review & editing, Funding acquisition, Methodology, Supervision.
